# Intensified glycemic control by HbA1c for patients with coronary heart disease and Type 2 diabetes: a review of findings and conclusions

**DOI:** 10.1186/s12933-023-01875-8

**Published:** 2023-06-22

**Authors:** Jingyang Chen, Dong Yin, Kefei Dou

**Affiliations:** 1 Cardiometabolic Medicine Center, Fuwai Hospital, National Center for Cardiovascular Diseases, Chinese Academy of Medical Sciences and Peking Union Medical College, Beijing, 100037 China; 2 Cardiometabolic Medicine Center, Department of Cardiology, State Key Laboratory of Cardiovascular Disease, National Center for Cardiovascular Diseases, Fuwai Hospital, Chinese Academy of Medical Sciences and Peking Union Medical College, Beijing, 100037 China

**Keywords:** HbA1c, Coronary heart disease, T2DM, Intensified glycemic control, Curvilinear correlation, HbA1c variability, GLP-1 RA, SGLT2i, Haptoglobin phenotype

## Abstract

The occurrence and development of coronary heart disease (CHD) are closely linked to fluctuations in blood glucose levels. While the efficacy of intensified treatment guided by HbA1c levels remains uncertain for individuals with diabetes and CHD, this review summarizes the findings and conclusions regarding HbA1c in the context of CHD. Our review showed a curvilinear correlation between regulated level of HbA1c and therapeutic effectiveness of intensified glycemic control among patients with type 2 diabetes and coronary heart disease. It is necessary to optimize the dynamic monitoring indicators of HbA1c, combine genetic profiles, haptoglobin phenotypes for example and select more suitable hypoglycemic drugs to establish more appropriate glucose-controlling guideline for patients with CHD at different stage of diabetes.

## Introduction

Cardiovascular diseases (CVDs), encompassing ischemic heart disease, stroke, heart failure, peripheral arterial disease, and an array of other cardiac and vascular ailments, constitute the leading cause of mortality globally, greatly impairing individuals’ quality of life [1, 2]. Ischemic heart disease, also referred to as coronary heart disease (CHD), stands as the prevailing manifestation of CVD on a global scale. As the report by the Global Burden of Disease Study, the worldwide burden of CHD amounted to 197 million cases, culminating in 9.14 million fatalities in the year 2019 [3]. According to the 2023 update on heart disease and stroke statistics carried out by the American Heart Association (AHA), approximately 20.5 million individuals aged 20 years and above in the United States are affected by CHD, indicating a prevalence rate of approximately 7.1% [4]. In China, CHD emerged as the second primary cause of mortality in 2016 and exhibits a concerning trend towards potentially becoming the leading cause of death in the near future [5].

**Fig. 1:**
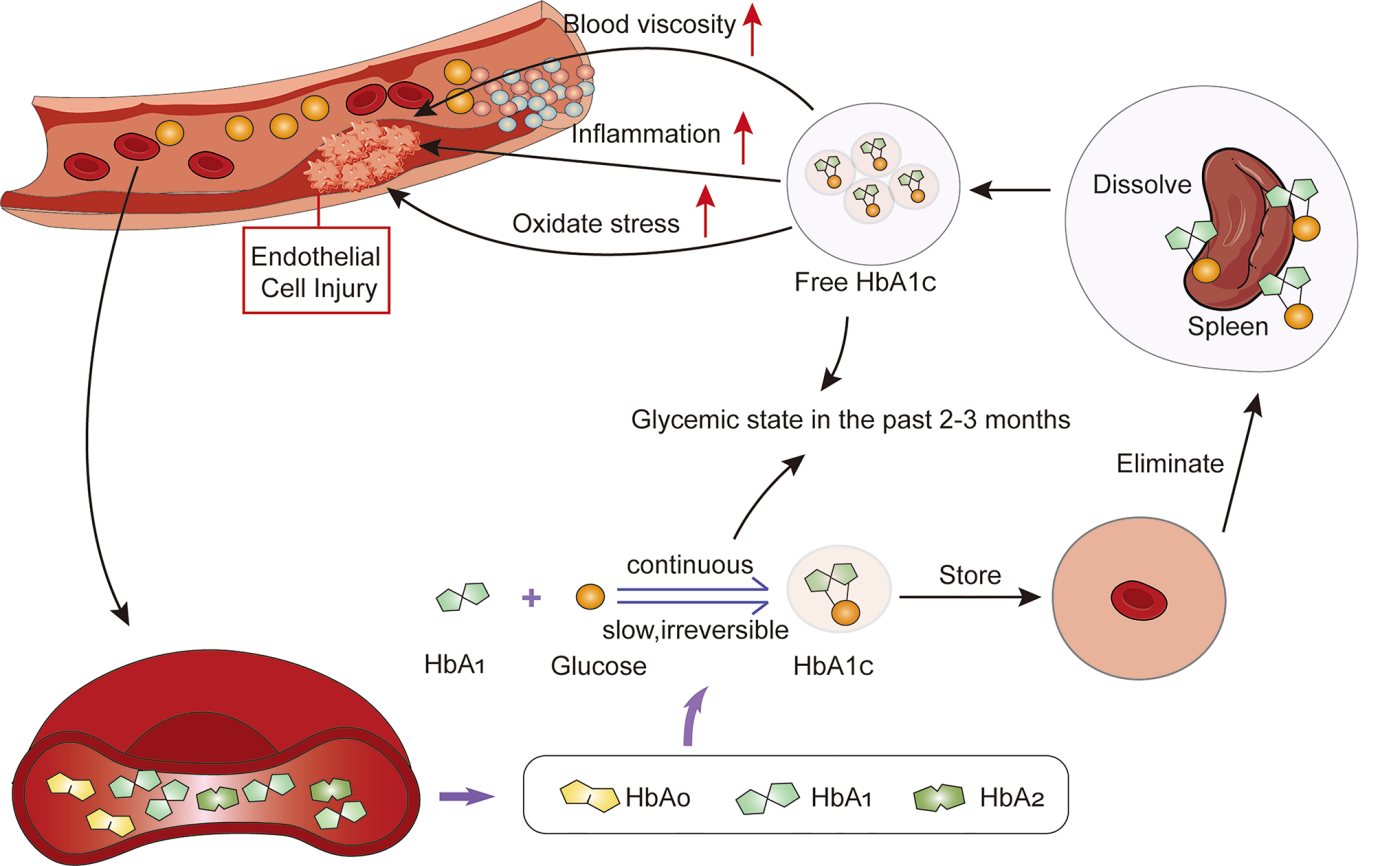
The formation of HbA1c and its role in mirroring glycemic state and driving cardiovascular damage **Legend**: There are three subgroups of hemoglobin, with HbA1 being the majority. HbA1a, HbA1b, and HbA1c are three types of glycoproteins, among which HbA1c accounts for the most and has a stable structure. The reaction to form HbA1c is continuous, slow, and irreversible, which makes HbA1c an indicator of glycemic state in the past 2–3 months and unaffected by short-term blood glucose levels. HbA1c is mainly dissolved into plasma by spleen. Free HbA1c can increase inflammation, oxidative stress, and blood viscosity to drive endothelial cells injury, which causes cardiovascular damage

Type 2 diabetes (T2DM) is the well-known economic and social burden for today’s real world [6]. It is widely recognized that T2DM fosters the progression of atherosclerosis, a pathological mechanism that can significantly contribute to the development of cardiovascular disease, this pathophysiological process largely depends on the long-term blood glucose level. Until the discovery of glycosylated hemoglobin, researchers had not identified a suitable marker to reflect the long-term glycemic level in individuals. In 1958, Huisman and Mayering classified hemoglobin (Hb) into three subgroups: HbA0, HbA1, and HbA2, with HbA1 comprising the majority [7]. In 1958, Allen et al. further subdivided HbA1 into three subtypes, naming them HbA1a, HbA1b, and HbA1c [8]. They discovered that HbA1c accounts for approximately 70% of HbA1 as exhibits relatively stable structure(8). It was not until 1968 that Brookchin and Gallop uncovered the nature of these subtypes as glycoproteins, revealing that HbA1c results from the reaction between HbA1 and glucose [9]. Later, in 1969 Rahbar et al. discovered that HbA1c was increased in the blood of patients with diabetes [10]. In 1976, Koening and Cerami introduced HbA1c as a clinical factor in monitoring glycemic control in patients with diabetes for the first time [11]. In Figure 1, we depicted the process of HbA1c formation and its role in contributing to cardiovascular damage. Due to the continuous, gradual, and irreversible nature of the non-enzymatic reaction leading to the formation of glycosylated hemoglobin, the concentration of HbA1c is primarily influenced by long-term blood glucose levels rather than short-term fluctuations that may be affected by immediate factors such as exogenous insulin administration or temporary glucose intake [12, 13]. HbA1c serves as a reflection of the glycemic status over a span of approximately 2 to 3 months [14]. As red blood cells undergo destruction by the spleen, HbA1c is released into the bloodstream. Apart from indicating long-term blood glucose level, free HbA1c can increases C-reactive protein [15], oxidative stress [16, 17], and blood viscosity [18]. These processes collectively contribute to the development of cardiovascular diseases by causing damage to the endothelial cells lining the blood vessels [19]. HbA1c has convenient and practical clinical operation value [20]. The HbA1c level in patients has demonstrated predictive value in various diabetes-related complications and has gained widespread recognition as an indicator for glycemic control in individuals with T2DM. However, its effectiveness lacks sufficient persuasiveness or consistency in patients with concomitant cardiovascular diseases, particularly CHD.

Therefore, our endeavor involved summarizing the divergent perspectives on the use of HbA1c as a predictor of clinical outcomes in patients with both T2DM and CHD. We also examined the inconsistent advantages of intensified treatment targeting HbA1c reduction in this specific population. Additionally, we cited potential factors that might contribute to this lack of consistency.

## Methods

Our review firstly investigated the inconsistent benefits of intensive treatment for patients with both T2DM and CHD, specifically focusing on the population receiving PCI or CABG. In this section, the search terms used were “HbA1c” OR “Glycosylated Hemoglobin” AND “coronary artery disease” OR “CHD” AND “T2DM” OR “Type 2 diabetes mellitus” AND “intensified glycemic control” OR “intensive glycemic control” AND “PCI” OR “Percutaneous Coronary Intervention” AND “CABG” OR “Coronary Artery Bypass Grafting”. We then focused on the curvilinear correlation between HbA1c and CVD risk reduction. The search terms in this section included “J-shaped” OR “U-shaped” OR “curve relationship” AND “intensive glycemic control” OR “intensified glycemic control”. We further listed the factors that might impact the inconsistent benefits of intensified glycemic control. In this section, the search terms were “HbA1c variability” AND “haptoglobin phenotype” OR “HP phenotype” AND “GLP-1 RA” OR “Glucagon-like peptide 1 receptor agonist” AND “SGLT2i” OR “Sodium-glucose cotransporter 2 inhibitors” AND “MACE” OR “major adverse cardiovascular events” AND “hypoglycemia”. There were no restrictions on study type, language, or time. We utilized the electronic databases PubMed and Web of Science. Two authors (Jing-yang Chen and Dong Yin) retrieved the full texts of these studies and assessed them for eligibility. Disagreements were resolved through discussion.

## The inconsistent benefits of intensified treatment

Typically, the HbA1c level in a human body should be maintained within the range of 4–5.7%. When healthcare professionals implement intensified glycemic control, they are referring to interventions such as lifestyle modifications and medication regimens that aim to reduce a patient’s HbA1c level to a range like that of a healthy individual, which is generally below 6.0–7.0% [21]. Poor glycemic control is closely related to severe endothelial dysfunction, which exacerbates the process and outcomes of atherosclerosis [22]. As a long-term indicator of blood glucose levels, HbA1c is also increasingly recognized for its role in chronic complications of diabetes such as cardiovascular events. Undoubtfully, maintaining optimal blood glucose control is imperative for patients, and several meta-analyses have demonstrated that in individuals with type 2 diabetes, each 1% elevation in HbA1c is linked to an approximate 13% increase in the risk of cardiovascular events [23, 24]. Controlling HbA1c level < 7.0% yields significant benefits of attenuating the advancement of coronary artery calcification, thereby reducing the incidence of cardiovascular diseases in patients [25, 26]. However, there is still debate about whether T2DM patients need more strict blood glucose control, that is, intensified treatment [27]. Several clinical guidance recommend a target HbA1c level below 7.0% or 6.5% in the management of diabetes [28–30], while the American Diabetes Association guideline recommends that a less strict control target (HbA1c level < 8.0%) for patients with microvascular or macrovascular complications can also bring benefit [31]. Although further intensification of blood glucose control (HbA1c level < 7.0%) may significantly reduce the risk of softer but clinically important endpoints such as coronary artery revascularization and hospitalization for unstable angina, this strict control increases the risk of hard clinical endpoints (such as mortality and non-fatal stroke) [32].

### Previous result for intensified treatment based on HbA1c level

It seems that no matter for short-term (≤ 3–5 years) or long-term (≥ 5 years) benefit, lowering HbA1c level fails to bring consistent result. Generally, most of research approve the short-term benefit of intensified HbA1c control. The PROactive study found that for T2DM elder patients (≥ 70 years old) with a history of macrovascular disease, intensified glycemic control (lowering HbA1c 0.8% in average) significantly reduced the risk of MACEs and all-cause mortality [33]. The ADVANCE study showed that participants who received intensified glycemic control (HbA1c level < 6.5%) had a significantly lower risk of combined macro- and microvascular complication (18.1% vs. 20.0% with standard control; P = 0.01), but the result is mainly driven by the decreased risk of microvascular outcome and no significant effect was found on the decreased risk of macrovascular complication or death from cardiovascular causes [34]. The VADT 5.6-year followed-up study showed that intensified glycemic control did not significantly reduce the risk of MACEs (hazard ratio, 0.88; 95% CI, 0.74 to 1.05; P = 0.14) and all-cause mortality (hazard ratio, 1.07; 95% CI, 0.81 to 1.42; P = 0.62) in T2DM patients [35]. The ACCORD study also found that although the incidence of non-fatal myocardial infarction (MI) was reduced during the 3.5-year follow-up period (hazard ratio, 0.76; 95% CI, 0.62 to 0.92; P = 0.004) in patients who received intensive treatment to target normal glycated hemoglobin levels, there seemed to be no significant change in the risk of MACEs (hazard ratio, 0.90; 95% CI, 0.78 to 1.04; P = 0.16) [36]. The ACCORD study found that the 3.5-year mortality rate even increased (hazard ratio for cardiovascular cause, 1.35; 95% CI, 1.04 to 1.76; P = 0.02; hazard ratio for any cause, 1.22; 95% CI, 1.01 to 1.46; P = 0.04), which drove the study terminate the intensive treatment for safety reason and turned the patients in intensive treatment group to follow a mean of 1.2 years of standard glycemic therapy [36, 37]. When it comes to long-term benefit, there is much more controversy. Tian et al. declared that the benefit concluded from the ADVANCE study was unrelated to differences in baseline HbA1c levels among patients and could reflect the long-term benefits to some extent [38]. In the 10-year follow-up of the VADT study, although the risk of first-onset MACEs was significantly reduced in patients who received intensified treatment (hazard ratio, 0.83; 95% CI, 0.70 to 0.99; P = 0.04), the cardiovascular death or all-cause death rate was not significantly different from that of the conventional treatment group (P = 0.42 and 0.54, respectively) [39]. It is noteworthy that during the 10–15 year follow-up period of the VADT study, the beneficial effects observed in the intensified treatment group were no longer evident when patients were transitioned to conventional treatment with a target HbA1c level below 9.0%, this phenomenon may be attributed to the lack of “metabolic memory” in such patients [40].

### The uncertain benefit for patients underwent PCI/CABG

The uncertainty of the benefits of intensified treatment is also reflected in studies of percutaneous coronary intervention (PCI) patients, with inconsistent conclusions mainly regarding the timing and intensity of glycemic control. Corpus et al. demonstrated that patients who achieved optimal glycemic control (HbA1c level ≤ 7.0%) prior to undergoing PCI experienced significantly lower risks of target vessel revascularization, cardiac rehospitalization, and recurrent angina compared to those with poor glycemic control [41]. This finding was consistent with the results of Hwang et al’s [42]. For patients with diabetes receiving stent implantation, intensive glycemic control (HbA1c level ≤ 7.0%) during follow-up can significantly reduce the risk of MACEs (adjusted hazard ratio, 2.1; 95% CI, 1.10 to 3.95; P = 0.02) [43]. In contrast, Ike et al. reported that while the clinical outcomes were more favorable in the group with HbA1c levels < 6.9% compared to the group with HbA1c levels ≥ 6.9%, a multivariable analysis did not reveal a significant correlation between the endpoint of MACEs and the preoperative HbA1c level [44]. They suggested that differences in baseline characteristics resulted by glycemic control may have created the “false association“ [44]. Furthermore, Park et al’s study demonstrated that intensified glycemic control did not show a reduced risk of MACEs compared to relaxed control (HbA1c level ≥ 8.0%) (P = 0.672) [45]. It should be noticed that the above studies only measured a single HbA1c measurement before or after procedure, which only reflects partial glycemic control during the follow-up period after PCI. We propose that to assess the impact of intensified hypoglycemic treatment, it may be necessary to optimize the measurement of HbA1c indicators. This can be achieved by improving the understanding of HbA1c variability and implementing scientific stratification of patients.

The discrepancy is also evident in patients who underwent coronary artery bypass grafting (CABG). It is noticeable that many studies only focused on preoperative HbA1c level, which mirrors the glycemic control before CABG. Pre-surgery HbA1c level either fails to predict in-hospital mortality or acute postoperative adverse events (within 30 days) [46, 47]. But it was found to be a significant predictor of worse long-term survival after surgery [46, 48, 49]. Cmolik et al. revealed that pre-operative HbA1c levels > 8% indicated higher mortality rate as well as increased risk of MI [50]. DiScipio et al. found that death risk increased by 13% for every 1% increase in HbA1c (adjusted hazard ratio, 1.13; 95% CI, 1.07 to 1.19; P < 0.001) [46]. In a prospective study involving 549 patients with T2DM who underwent CABG, it was observed that maintaining a preoperative HbA1c level within the range of 6.1-7.0% yielded the best outcomes in terms of management [51]. Higher HbA1c level (> 8.0%) was associated with increased risk of MACEs, while HbA1c level below 6.0% was linked to elevated risk of mortality (hazard ratio, 2.41; 95% CI, 1.01–5.74) [51]. We agreed that the increased risk of acute adverse outcomes may be due to the climbing risk of hypoglycemia when treating with intensive control. Interestingly, Zheng et al’s study found that controlling blood glucose < 7.8 mmol/L after procedure predicted severer in-hospital all-cause mortality and major cardiovascular complications (adjusted odds ratio, 2.22; 95% CI 1.18–4.15; P = 0.01) [52]. Regrettably, there is a lack of comprehensive research on postoperative HbA1c control and long-term effect in patients with T2DM undergoing CABG are relatively scarce.

### The curvilinear correlation between HbA1c and CVDs risk reduction

In 1998, Bonora et al. conducted a study that revealed a U-shaped or J-shaped relationship between serum insulin levels and coronary heart disease in the general population [53]. Interestingly, there appears to be a curvilinear trend in the relationship between HbA1c levels and therapeutic benefit. We have collected and organized the trend patterns and corresponding cut-off points mentioned in recent literature, which are presented in Table 1.

**Table 1:** Trend patterns and optimal cut-off points of HbA1c level for patients with T2DM and CHD

Article	Population	Followed-up year(s)	outcome	Curve type	Truning point/area of HbA1c	Citation
Choi et al.	Patients with AMI undergoing PCI with diabetes mellitus had ≥ 3 HbA1c measurement	6.2	All-cause mortality	J-shaped	6.5 to 7.0%	[61]
Östgren et al.	Patients with T2DM aged ≥ 35 using glucose-lowering agents	4.8	Cardiovascular event All-cause mortality cardiovascular mortality	J-shaped	6.8% in oral-agents group 7.3% in insulin group	[62]
Li et al.	Patients with gout from the UK Biobank.	7.3	Cardiovascular events	Quasi J-shaped	5.0%	[63]
Plakht et al.	Diabetic patients underwent non-fatal AMI	10	All-cause mortality	U-shaped	6.5 to 7.0%	[54]
Funamizu et al.	Patients with preprocedural HbA1c values underwent PCI	10	Cardiovasular mortality Sudden death	U-shaped	7.0 to 7.5%	[55]
Wang et al.	Patients with CHD	1	MACEs TLR	U-shaped	5.9 to 6.8%	[56]
Yang et al.	ACS patients with T2DM and underwent PCI	5	MACEs	U-shaped	6.5 to 7.5%	[57]
McAlister et al.	Patients with T2DM and atherosclerotic vascular disease	3	hFH All-cause death Cardiovascular death Non-HF Cardiovascular outcomes Worsening kidney function Severe hypoglycemic events	U-shaped	7.0%	[58]
Liu et al.	Patients with CHD	4	Long-term all-cause death	U-shaped	5.7 to 6.7%	[59]
Baber et al.	Patients undergoing PCI	1	MACEs	U-shaped	6.1 to 7.0%	[60]

AMI: Acute myocardial infarction; T2DM: Type 2 diabetes; MACEs: Major adverse cardiac events; TLR: Target lesion revascularization; CHD: Coronary heart disease; ACS: Acute coronary syndrome; PCI: Percutaneous Coronary Intervention; HF: heart failure; hHF: HF hospitalization

Whether it follows a U-shaped [54–60] or a J-shaped [61–63] curve, it is clear that HbA1c management should aim to stabilize levels at an appropriate intermediate range. However, it is not yet clear whether this linear relationship is genuine or if it is influenced by other non-glycemic factors such as age, duration of diabetes, follow-up time or drug treatment. For example, a similar trend may not be easily observed with a follow-up time that is too short (30 months) [32]. Östgren et al. showed different cut-off points for T2DM patients using insulin or oral drugs [62]. The utilization of sulfonylureas for the purpose of intensive HbA1c control did not confer additional advantages in terms of macrovascular disease [64]. This finding suggests that the selection of sulfonylureas, characterized by their hypoglycemic properties and potential cardiac toxicity, may counterbalance the beneficial effects of glycemic control on long-term outcomes [64]. Furthermore, Ghouse et al. expanded on the findings of Choi et al [61]. by providing additional insights, revealing a J-shaped relationship between mean HbA1c level and mortality risk only in elderly patients (≥ 65 years old) with a diabetes duration of ≥ 5 years [65]. It’s worth noticing that mortality rates are increased both for patients with HbA1c ≥ 8.0% due to cardiovascular mortality, and for those with HbA1c ≤ 6.5% due to both cardiovascular and non-cardiovascular causes of death [61]. This phenomenon may be explained by the increased risk of severe hypoglycemic events associated with intensified treatment [38]. Lee et al’s study found that severe hypoglycemic events are associated with CHD, all-cause mortality, and cardiovascular mortality [66], this may be due to poor physiological metabolic reserve capacity in patients with low HbA1c levels [61, 67]. Raghavan et al. found that HbA1c <6% significantly increase mortality rates compared to levels<6.9%, while levels ≥ 7% only affects risk of MI in patients with obstructive CHD [68]. It’s possible that the severity of coronary artery lesions and cardiac function exacerbates the impact of hypoglycemia on mortality, suggesting that HbA1c may not be a reliable therapeutic indicator for patients with such severe lesions.

The uncertainty surrounding the benefits of different studies makes it challenging to manage patients with T2DM in clinical settings. Factors such as the duration of diabetes, preexisting macrovascular disease, occurrence of hypoglycemic events and significant complications should be considered when deciding on the appropriate glycemic management strategy [69]. It is essential to tailor these criteria to each patient, taking into account individual differences in addition to diabetes-related factors. Improving the efficacy of HbA1c as a biochemical indicator is also an area of concern that requires further attention.

## The potential factors for optimizing glycemic control further

HbA1c, also known as glycated hemoglobin, has emerged as a potentially valuable biomarker of cardiovascular risk and prognosis in patients with diabetes. However, the existing body of evidence is currently insufficient to establish consistent findings on this matter. We surmise that researchers’ inability to comprehensively understand the exact glucose fluctuations of patients throughout the follow-up period leads to incomplete or inconsistent findings. We hypothesize that three potential factors may contribute to the inconsistency of the results: variability in HbA1c levels, the phenotype of haptoglobin and the selection strategy for hypoglycemic drugs.

### HbA1c variability

Blood glucose fluctuation is an emerging metabolic index that has gained attention in recent years. It can be categorized into two types: long-term blood glucose fluctuation and short-term blood glucose fluctuation. The latter can be evaluated by continuous blood glucose monitoring (e.g., blood glucose fluctuations at 2 h after meals) or blood glucose levels during hospitalization to predict poor cardiovascular outcomes [70–73]. The former has been shown to be a better predictor of chronic complications in patients with diabetes, such as macrovascular and microvascular lesions, providing valuable prognostic guidance [74–77]. For patients with diabetes undergoing PCI, long-term blood glucose variability has been associated with an increased incidence of perioperative MI and MACE within 6 months after surgery [78]. Furthermore, unstable long-term blood glucose fluctuations can result in chromatin recombination, leading to persistent vascular dysfunction, which may explain why some T2DM patients with HbA1c control are still at a higher risk of MACEs [79].

HbA1c is widely recognized as a reliable measure of long-term blood glucose variability. In recent years, the concept of Visit-to-visit HbA1c variability (VVV of HbA1c) has been proposed by some scholars to track HbA1c fluctuation at different treatment periods [80–82]. “Visit-to-visit HbA1c variability” refers to the variation in the measurement of HbA1c over multiple medical visits, commonly used measures of HbA1c variability include Average HbA1c, standard deviation of HbA1c (HbA1c-SD) after multiple measurements of individuals, and coefficient of variation of HbA1c (HbA1c-CV). Initially, research on the relationship between HbA1c variability and diabetes complications primarily focused on microvascular complications in type 1 diabetes, without extending to macrovascular outcomes in type 2 diabetes [83]. But in recent years, there has been an increasing number of studies aimed at determining the significance of HbA1c variability in patients with T2DM. We present several applications of HbA1c variability in predicting the risk and prognosis of CAD in Table 2. In addition to HbA1c-SD and HbA1c-CV, another measure of HbA1c variability called the HbA1c variability score (HVS) has been introduced. HVS is calculated as the proportion of HbA1c fluctuation from the last visit to the next visit divided by the total number of follow-up measures and multiplied by 100 [84, 85]. Individuals with an HVS of 60% or higher exhibit a higher risk compared to their counterparts and show a stronger association with cardiovascular outcomes [86]. This indicates that HVS serves as a more reliable predictor of cardiovascular events in patients with diabetes compared to a single HbA1c value, as it reflects less stringent and controlled glucose management.

**Table 2:** Applications of HbA1c variability in prediction of CVD outcomes for patients with T2DM

Article	Inclusion	Parameters for HbA1c variability	Follow-up time/measurement times	Related outcomes	Reference
Yang et al.	Patients with T2DM accepted successful stent implementation through PCI	HbA1c-CV HbA1c-SD VIM	12 months/ ≥ 3/3months	In-stent restenosis	[80]
Sun et al.	Patients with T2DM	HbA1c-CV	4.8 years/ 6 times	Macrovascular and microvascular events	[93]
Wan et al.	T2DM Patients without prior CVDs	HbA1c-Mean HbA1c-SD	7.4 years/ 3.2 times	CVDs All-cause mortality	[91]
Forbes et al.	Patients ≥ 70 years old with diabetes	HbA1c-Mean HbA1c-SD	5 years/ ≤ 6 times	All-cause mortality	[84]
Penno et al.	Patients with T2DM	HbA1c-Mean	2 years / 3–5 times	CVDs	[94]
Hirakawa et al.	Patients in ADVANCE Trial with intensified treatment	HbA1c-SD	1 year / 5 times	Cardiovascular events	[92]
Yang et al.	Patients with T2DM underwent CARTs	HbA1c-SD HbA1c-CV adj-HbA1c-SD	5.5 years / ≥ 6 times	Cardiovascular autonomic neuropathy	[95]
Shen et al.	Patients with T2DM	HbA1c-SD HbA1c-CV adj-HbA1c-SD	2 years/ ≥ 4 times	CHD and stroke	[81]
Lee et al.	Patients with T2DM with preserved renal function	HbA1c-SD	6 years/ ≥ 3 times	Hospitalization for CVDs	[96]
Yang et al.	Patients with T2DM without CHD history	HbA1c-SD HbA1c-CV adj-HbA1c-SD	973 days/ ≥ 4 times	Subclinical coronary atherosclerosis	[97]

VIM: Variability independent of the mean (100 × SD/mean·β, β: the regression coefficient based on natural logarithm of SD on natural logarithm of mean of the study population); CV: Coefficient of variation; SD: Standard deviation; Adj-HbA1c-SD: Adjust for the number of HbA1c assessments (HbA1c-SD / √[n / (n − 1)]); CVDs: Cardiovascular diseases; CHD: Coronary heart disease; T2DM: Type 2 diabetes; PCI: Percutaneous coronary intervention; CARTs: Cardiovascular autonomic reflex tests

For patients who undergo PCI, the post-procedural maintenance phase is an extended and protracted period. During this phase, long-term complications, such as restenosis, frequently occur after a significant duration. Therefore, we contend that the prediction of prognosis should be based on dynamic changes in HbA1c levels during the follow-up. Yang et al. discovered that the HbA1c variation rate (CV), standard deviation (SD), and VIM levels were closely related to the risk of stent restenosis in PCI and stenting patients, subjects with HbA1c ≤ 7% exhibited more severe restenosis when HbA1c-CV was high, as compared to subjects with HbA1c > 7% [80]. This conclusion may be attributed to patients’ poor postprandial blood glucose control [87]. Jiang et al. conducted a study that suggested postprandial blood glucose levels could potentially be utilized as a screening parameter for early-stage coronary artery disease in patients with coronary artery disease [88]. Further relevant studies are required to validate its value in the postoperative prediction of PCI patients.

The dynamic observation of HbA1c levels in patients can better demonstrate the significance of HbA1c in the management of cardiovascular complications. Nonetheless, it is crucial to note that the predictive effectiveness of different indicators may differ. Critchley et al’s study revealed that HbA1c-CV had a significant dose-response relationship with all-cause mortality in patients with diabetes compared to traditional average HbA1c, whereas average HbA1c exhibited more precise predictive value in predicting the risk of mortality and hospitalization in individuals with coronary artery disease [89]. Ceriello et al’s study indicated that the prognostic significance of HbA1c-CV in T2DM patients regarding cardiovascular complications could be influenced by the initial level of HbA1c fluctuation [90]. This association appears to be more pronounced in cases where patients exhibit higher levels of blood glucose variability, indicating unstable glycemic control [90]. Else, Wan et al. revealed that the predictive effect of mean-HbA1c and HbA1c-SD for CVDs may be inversely associated with age, with a 28% higher risk per 1% increase in HbA1c variability in the age group 45 to 54 years, whereas only a 14% higher risk in the 75–84 age group [91]. By the way, considering the curve relationship described [54–63], similar conclusions have not been generally obtained in most HbA1c variability studies. Hirakawa et al. found that in the intensified group of the ADVANCE Trial, a decrease in VVV of HbA1c was consistently associated with a lower risk of vascular events [92]. Forbes et al. showed that a J-shaped relationship between HbA1c-mean and all-cause mortality, with significant increases with HbA1c-Mean values greater than 8% and less than 6% [84]. This may be account for the inclusion of type I diabetes mellitus patients and the study didn’t specialize CVD events.

Regrettably, at present, there is no universally recognized “golden standard” to quantify the variability of HbA1c, and there exists a lack of consensus regarding its potential clinical significance. Therefore, it is essential to determine an accurate, efficient, feasible, and widely recognized classification of the various HbA1c measurements as soon as possible to further promote its importance in prevention, prognosis, and treatment guidance. Additionally, further randomized controlled trials are required to evaluate and verify the predictive value of HbA1c variability in the risk of cardiovascular events in patients with pre-diabetes and patients without diabetes.

### Haptoglobin phenotype

Haptoglobin (Hp) is an acidic glycoprotein, which widely exists in human and various mammalian species and mainly synthesized in the liver. The main function of Hp is to combine with serum hemoglobin to form a stable Hp-Hb complex [98]. Briefly, Hp helps prevent oxidative damage by cleaning abnormal Hemoglobin, including HbA1c [99, 100]. Hp acts an essential role in HbA1c clearance. The frequency of Hp phenotypes varies with ethnicity and geographical location [101], and it is the phenotypic differences that determine the functional variation [102]. The Hp phenotype plays a crucial role in determining the ability of cleaning HbA1c, determines the tolerance of body to HbA1c concentration [103]. We supposed that differentiating Hp phenotype of individuals can help us to further develop the maneuver of glycemic control.

Hp has two genotypes, Hp1 and Hp2, with allele frequencies of approximately 40% and 60% worldwide respectively [101]. There are three common phenotypes: Hp1-1, Hp1-2, and Hp2-2. The Hp1-1 genotype is the predominant genotype observed in South America, whereas the Hp2-2 genotype is more commonly found in Southeast Asia [101]. These three phenotypes can be easily distinguished molecularly based on different molecular weight and structure. The Hp2-2 phenotype produces larger and more circular proteins. This structural characteristic has been linked to intravascular oxidation and was previously considered to be a prominent phenotype selected for during the early stages of human evolution, particularly in relation to infectious diseases [98]. However, this particular phenotype has been associated with an increased risk of complications in certain non-infectious inflammatory diseases [104], especially the diabetic macro- and micro-vascular complications [105].

**Fig. 2:**
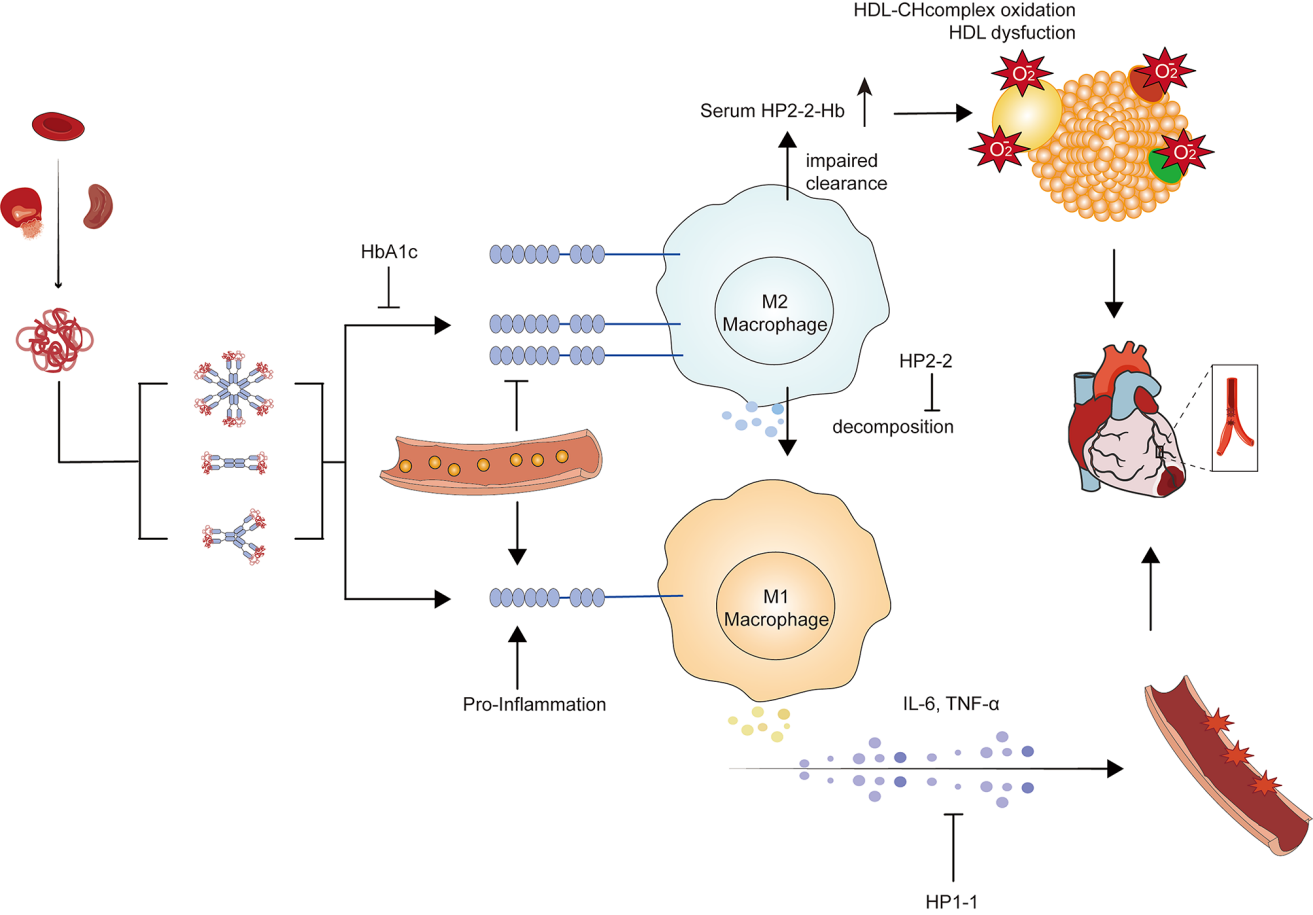
The mechanism of Hp phenotype to cause cardiovascular diseases **Legend**: Normally, the Hp-Hb complex is mainly captured by the scavenger receptor CD163 of M2 macrophages and then decomposed. Hp2-2 phenotype can impair the function of clearance, Hyperglycemia can further reduce the number of CD163 on the cell membrane while HbA1c can further hamper the binding between Hp-Hb and CD163. Consequently, the Hp2-2-Hb complex induces the dysfunction of HDLc and leads the oxidation to damage of blood vessel. Hyperglycemia can also induce the expression of CD163 on M1 macrophages and convert the silent clearance into an inflammatory process (Hp2-2 further induces the long-lasting inflammation), resulting vessel damage

The Hp-Hb complex is primarily captured by the scavenger receptor CD163, which is predominantly expressed on M2 macrophages, and subsequently undergoes decomposition [106]. Though Hp2-2-Hb complexes do show a 10-fold higher affinity to CD163 compared to Hp1-1-Hb complexes [107], the cleaning function of Hp2-2 phenotype is relatively poor, and the presence of HbA1c further attenuates its binding ability [108]. Hyperglycemia can exacerbate the reduction in the number of CD163 receptors on the cell membrane, leading to an elevation in the circulating levels of Hp2-2-Hb complexes within a hyperglycemic environment [108–110]. Compared to non-Hp2-2-Hb complex, the Hp2-2-Hb complex can bind to high-density lipoprotein (HDL), tether Hb to HDL and then enhance the generation of oxidized cholesterol and other related components within HDL [111]. As a result, the impaired function of HDL-cholesterol leads to the oxidation of various lipid and protein substrates, ultimately contributing to blood vessel damage [112–114], accelerates the progression of atherosclerosis [108–110], and promotes the occurrence of coronary heart disease [115]. Else, hyperglycemia and pro-inflammation can also induce the expression of CD163 on M1 macrophages and shift the silent clearance into an inflammatory process, regardless the phenotype of Hp (Hp2-2 can promote much long-lasting proinflammatory cytokines release than Hp1-1) [116]. We present the classification of Hp phenotypes and two mechanisms by which Hp phenotypes contribute to cardiovascular disease in Fig. 2.

Consistent with the result of in vitro study, several studies have also shown that diabetes interacts with Hp phenotype and the association of Hp 2-2 with CVD is observed only in population with diabetes [117–119]. In 2013, Cahill et al. found that Hp2-2 represents a higher CHD risk in the Nurses’ Health Trial, and this association only showed significantly in the population with HbA1c level ≥ 6.5% [120]. Later, Cahill et al. further developed this conclusion in another study, revealing that compared with HbA1c level < 6.5%, the risk rate of CHD for HbA1c level ≥ 6.5% is significantly elevated in patients with Hp 2-2 genotype over full follow-up and the first half of follow-up, while individuals with Hp2-2 phenotype and HbA1c level < 6.5% didn’t have significant increased CHD risk [121]. It means that the Hp 2-2 genotype may identify the susceptibility of CHD in individuals with hyperglycemia, especially in short-term period. However, there are also some inconsistent results. Pechlaner et al’s Bruneck Study didn’t find an increased CVD risk in Hp 2-2 people with elevated HbA1c after age- and sex-adjustment [122]. De Bacquer et al’s study demonstrated that among patients without diabetes or just with pre-diabetes, the Hp1-1 phenotype was associated with an increased risk of cardiovascular disease-related mortality, rather than Hp2-2 [123]. Intriguingly, in 2004, the study on Framingham offspring (European decent) found no significant cardiac risk between the different Hp phenotypes in general population, but subgroup analysis found a disparate result, demonstrating an increased risk of incident CHD in Hp 1-1 patients with diabetes and a decreased risk of CHD mortality in Hp 1-1 patients without diabetes [124]. A meta-analysis conducted by Gurung et al. focusing on Chinese patients indicated a similar conclusion that individuals with non-Hp 2-2 were at a higher risk of AMI [125], which aligns with the results of Wang et al’s study on Chinese T2DM population [126] but contrary to the Cahill’s result which mainly focused on Native American population [120, 121]. Overall, we suppose that studies may yield different results due to variations in the study’s design, the ethnic makeup of the population, the age group and sex imbalance (menopausal status) [127], the survivorship bias (individual with the Hp2-2 phenotype have a shorter lifespan) [128] and the possibility of linkage between Hp and other unknown genes that may be involved in causing the disease. Furthermore, since Gurung et al’s study found no significant difference in LDLc levels among the different Hp phenotypes in included cohorts, it suggests that the mechanism by which Hp1 induces AMI may not be related to chronic atherosclerosis caused by traditional lipid oxidation [125].

The difference of Hp phenotype may explain the differences in efficacy of intensified blood glucose control tests among subjects of different regions and races [129]. Recently, researchers began to study the impact of Hp phenotype for intensified treatment. The association between Hp phenotype and outcomes after intensified/conventional therapy was investigated in a large randomized controlled trial by Carew et al., who showed that patients with Hp2-2 phenotype who received intensified therapy had less CHD and CVD outcomes, while the intensified treatment brought increased mortality risk for Hp1-1 patients [130]. However, this study has been challenged because the interaction p values for Hp phenotype and strict glycemic control on CVD outcomes did not reach significance in Carew et al’s study (the p values of CHD, CVD, non-fatal MI, fatal CVD and total mortality is 0.059, 0.126, 0.212, 0.253 and 0.110 respectively), the article failed to reject the null hypothesis of identical cardiovascular effects of intensified glycemic control in Hp2-2 and Hp1 carriers [131]. Although Carew et al. replied that limitation could be due to lack of power or to true lack of interaction, it can only be a hypothesis that for patients with Hp2-2 phenotype, maintaining a normal level of HbA1c may be more effective in reducing the risk of cardiovascular events. Undoubtedly, this hypothesis provides us with a novel perspective on the individualization and customization of glycemic treatment for diverse individuals, considering that Hp phenotype is an inherent characteristic of individuals. Further research is needed to support this prediction, we suppose that: (1) Due to the limited popularity of Hp phenotype detection, most studies have included relatively small populations. Building large-scale cohorts will be essential for further confirmation. (2) Given the contrasting results observed between Chinese and European populations, it is important to consider the influence of ethnicity and DNA polymorphism. Comparative studies across different ethnic groups can provide valuable insights. (3) With the widespread use of invasive treatments like PCI for cardiovascular events, it is important to investigate the potential benefits of intensified glycemic control specifically in Hp2-2/non-Hp2-2 individuals who have undergone such interventions.

### The drugs selection for intensive glycemic control

The heightened risk associated with intensive glycemic control, particularly in patients undergoing PCI or CABG, primarily stems from the potential occurrence of hypoglycemia. We propose that medications with minimal hypoglycemic risk should be prioritized as the preferred option for achieving intensive glycemic control in such patients. Considering the widespread utilization and low hypoglycemic risk, we particularly consider two sorts of newly developed hypoglycemic agents: Glucagon-like peptide-1 receptor agonists (GLP-1 RAs) and Sodium-glucose cotransporter 2 inhibitors (SGLT2is).

GLP-1 RAs represent a novel class of hypoglycemic drugs that operate by activating the GLP-1 receptor. These agents enhance insulin secretion in response to glucose levels, suppress glucagon secretion, and delay gastric emptying [132], reducing food intake through central appetite inhibition, thereby achieving a decrease in blood sugar levels [133].

Up to now, scientists have proven that some of GLP-1 RA plays a role in protecting patients with T2DM and CVD from MACEs, including liraglutide [134, 135], efpeglenatide [136], dulaglutide (attenuate the risk of MACEs and non-cardiovascular death but not the all-cause death rate) [137, 138]. There are clinical studies have demonstrated that the use of GLP-1 RAs leads to a reduction in the risk of major adverse cardiovascular events (MACEs) by effectively lowering HbA1c levels [139, 140]. But GLP-1 RAs are associated with a low incidence of severe hypoglycemia. A study conducted by Frandsen et al. found no significant increased rate of severe hypoglycemia among patients with T2DM and high cardiovascular risk who were treated with liraglutide alongside basal insulin compared to those without liraglutide treatment [141]. Similarly, a study examining Semaglutide yielded a similar conclusion, observing a non-increased incidence of hypoglycemia vs. sitagliptin, exenatide or insulin [142]. Wang et al. even discovered that the occurrence of hypoglycemia was notably lower in patients with T2DM who received dulaglutide compared to those treated with glargine [143].

Furthermore, a study conducted by Nathan et al. revealed that, when combined with metformin, liraglutide exhibited a significant capacity to decrease the risk of MACEs in patients with T2DM, in comparison to glargine, glimepiride, and sitagliptin [144]. As a recently developed GLP-1 RA, efpeglenatide monotherapy has exhibited noteworthy reductions in the risk of MACEs in patients diagnosed with T2DM [145]. In a study conducted by Ludvik et al., it was observed that tirzepatide, a novel dual glucose-dependent insulinotropic polypeptide and GLP-1 RA, exhibited superiority over titrated insulin treatment, showing greater reductions in HbA1c levels while maintaining a safety profile similar to other GLP-1 RAs [146]. These findings suggest that GLP-1 RAs may be suitable for achieving intensified glycemic control due to their improved effectiveness and satisfactory safety profile. The widespread use and promotion of GLP-1 RAs may potentially alleviate the contradictory effects of intensive glucose reduction observed in patients with T2DM and CHD. However, further research focusing on intensive glycemic treatment with GLP-1 RAs is required to validate this hypothesis.

SGLT2is, a novel class of medications used for diabetes management, operate by inhibiting the reabsorption of glucose in the kidneys. This mechanism results in an elevated excretion of excess glucose through the urine [147]. SGLT2is facilitate the elimination of glucose rather than enhancing its uptake. This distinctive attribute leads to a notable decrease in the risk of hypoglycemia, while still achieving similar reductions in HbA1c levels when compared to other glucose-lowering treatments [148].

Despite being primarily utilized for glycemic control in patients with T2DM, SGLT2is now have showed notable benefits in terms of heart protection and been recommended for patients with heart failure, irrespective of their T2DM status [149–151]. However, its benefit of reducing the risk of MACEs in patients with T2DM and CVDs remains a subject of controversy. Zinman et al. revealed that empagliflozin showed significantly reduction in the rates of death from cardiovascular causes but did not reduce the incidence of MI or stroke [152]. According to the findings conducted by Wiviott et al., dapagliflozin was associated with a lower rate of hospitalization for heart failure in patients with T2DM who had or were at risk for atherosclerotic cardiovascular disease(ASCVD) [153]. However, it did not demonstrate a significant reduction in the risk of MACEs for these patients [153]. In the meta-analysis conducted by Zelniker et al., it was observed that SGLT2is exhibited a reduction in MACEs specifically in patients with established ASCVD [154]. This analysis suggests that the benefit of SGLT2is in terms of reducing MACEs may be more prominent in specific patient populations. Furtado et al. later revealed that dapagliflozin specifically reduces risk of MACEs in patients with T2DM and previous MI, rather than patients without definite MI history [155]. McGuire et al. found that empagliflozin reduced the total burden of MACEs in patients with T2DM and ASCVD [156], it might be attributed to the ability of SGLT2is to enhance myocardial flow reserve and protect the heart from microvascular dysfunction [157]. Considering that a significant number of patients undergo PCI or CABG for acute or previous MI, we propose that SGLT2is could be a suitable treatment option for individuals with severe coronary artery disease. SGLT2is offer the advantage of reduced hypoglycemic risk, making them an appealing choice for this patient population.

Interestingly, while SGLT2is have been shown to significantly lower HbA1c levels, including HbA1c variability [158], it is noteworthy that the observed benefits of reducing MACEs with SGLT2is appear to be independent of their effect on lowering HbA1c [159, 160], showing possibility of SGLT2i to be a favorable choice for people with definite MI but without definite hyperglycemia to exert strict glycemic control. This finding differs from the mechanism by which GLP-1 RAs reduce the risk of MACEs [139, 140]. Further research is needed to figure out the exact mechanism of SGLT2i reducing MACEs risk.

Overall, based on our analysis, we propose that GLP-1 RAs are considered as an optimal class of hypoglycemic drugs for implementing safer intensive glycemic treatment in patients with T2DM, while SGLT2i may be a suitable option, particularly for patients with acute or previous MI, no matter with T2DM.

## Discussion

It is crucial to underscore the importance of continuous HbA1c measurement as it represents a dynamic and fluctuating indicator. Many studies have only conducted HbA1c measurements at baseline, which does not offer a complete monitoring of patients’ glycemic control. Long-term follow-up is necessary to fully comprehend the association between blood glucose levels, their stability, and the risk of coronary disease. Dynamic observation of HbA1c can provide a more comprehensive understanding of this relationship. However, for demonstrating the predictive value of acute or perioperative complications, short-term HbA1c measurements may be sufficient. Nonetheless, to obtain more objective data support, it is ideal to have multiple HbA1c measurements throughout the course of the study.

Further verification and improvement of the predictive efficiency of HbA1c variability is necessary. Though HbA1c variability shows weak and inconsistent associations with complications in the early stages of follow-up, its significance becomes more apparent over time [82]. We propose that the predictive ability of HbA1c is limited to a specific time window. Combining HbA1c with real-time dynamic blood glucose monitoring may enhance its predictive value during the early stages.

An alternative approach to improve the HbA1c prediction model is to use the hemoglobin glycosylation index (HGI), defined as the measured HbA1c minus predicted HbA1c, which combines fasting blood glucose (FBG) and HbA1c to overcome the incompatibility between HbA1c and average blood glucose [161]. The predicted HbA1c based on the average blood glucose level is calculated from the linear regression between FGB and HbA1c, which reflects the vascular health status of patients with glucose metabolism [162]. The combination of long-term and short-term blood glucose indices provides a more comprehensive monitoring effect. Furthermore, the prediction model of longitudinal fluctuation of patient HbA1c provides more accurate prediction than the average actual variability or coefficient of variation. The linear mixed effect model and Cox model combine longitudinal outcome information that is not constant in time with time-event data to capture the association between different factors and clinical endpoints [163]. Additionally, the development of artificial intelligence provides new opportunities for improving HbA1c prediction. Machine learning applied to predict patients’ blood glucose fluctuations and even HbA1c variability [164] can reduce the difficulty and cost of long-term patient follow-up, promoting more efficient individualized risk prediction and evaluation [165].

To improve the predictive efficacy of coronary heart disease events, individual HbA1c needs to be combined with genes. A Mendelian randomized analysis study on HbA1c genetically confirmed a causal relationship between HbA1c and CAD risk, driven not only by blood glucose but also by factors unrelated to blood glucose, such as age, sex, race, and red blood cell content [166, 167]. The variation in Hp phenotype has a substantial impact on the quality of patients’ coronary arteries and their susceptibility to cardiovascular complications. As individual Hp phenotype does not change with time and only needs to be measured once in a lifetime, the detection is also cost-effective and holds profound significance [168]. We suggest that it could be incorporated as a standard biomarker for the prognosis and treatment guidance of diabetic patients, complementing evidence-based medication strategies to optimize the intensity of hypoglycemic therapy. Additionally, we advocate for further research to explore the role of Hp phenotype and HbA1c level control in guiding treatment decisions for CHD patients, particularly in individuals without diabetes or pre-diabetes. This would contribute to advancing our understanding and promoting evidence-based practices in the field. Attention should be paid to Hp phenotypic specificity among different populations. Recently, a novel fourth phenotype, known as modified Hp2-1 (designated as Hp2-1 m), has been identified in the black population [169]. This newly discovered phenotype represents a distinct variation of haptoglobin and contributes to the expanding understanding of haptoglobin diversity within different ethnic groups. The potential association between the unique Hp2-1 m phenotype and cardiac macrovascular complications in the patients with diabetes in African population and whether it has value in guiding risk stratification and treatment should be further studied. What’s more, it is also important for medicators to select proper hypoglycemic drugs, we summarized two kinds of popular new-develop drugs: GLP-1 RA and SGLT2i. It seems that although both two can largely reduce HbA1c level of patients, the mechanisms by which GLP-1 and SGLT2i reduce the risk of MACEs are not the same, and there may even be some contradictions between them. We considered that GLP-1 RAs as an ideal type of hypoglycemic drugs for exerting safer intensive glycemic treatment in patients with T2DM, while SGLT2is are recommended for patients with acute or previous MI, irrespective of their glycemic status.

## Conclusion

This review highlights the uncertain benefits of intensified treatment based on HbA1c levels for diabetes patients with CVDs, indicating a curvilinear trend in the correlation between HbA1c level and therapeutic benefit. Promising advancements in glycemic management can be achieved by incorporating factors such as HbA1c variability, individual characteristics such as Hp phenotype, and the appropriate selection of hypoglycemic drugs. This comprehensive approach holds the potential to optimize treatment outcomes and improve overall patient care.

## Data Availability

Not applicable.

## Abbreviations

CVD: Cardiovascular disease
CHD: Coronary heart disease
T2DM: Type 2 diabetes
Hb: Hemoglobin
MACE: Major adverse cardiac event
PCI: Percutaneous coronary intervention
CABG: Coronary Artery Bypass Grafting
AMI: Acute myocardial infarction
MI: Myocardial infarction
TLR: Target lesion revascularization
ACS: Acute coronary syndrome
HF: Heart failure
hHF: HF hospitalization
VVV of HbA1c: Visit-to-visit HbA1c variability
CV: Coefficient variation
SD: Standard deviation
CARTs: Cardiovascular autonomic reflex tests
HVS: HbA1c variability score
Hp: Haptoglobin
HDLc: High-density lipoprotein cholesterol
LDLc: Low-density lipoprotein cholesterol
HGI: Hemoglobin glycosylation index
FBG: Fasting blood glucose
GLP-1 RA: Glucagon-like peptide-1 receptor agonist
SGLT2i: Sodium-glucose cotransporter 2 inhibitors
ASCVD: Atherosclerotic cardiovascular disease
